# Metastatic Hepatocellular Carcinoma Masquerading as an Expansile Osteolytic Lesion in Scapula: A Rare Case of Isolated Appendicular Skeletal Metastatic Involvement of Hepatocellular Carcinoma at Initial Presentation

**DOI:** 10.1055/s-0042-1757288

**Published:** 2022-10-31

**Authors:** Arun Ravi John, Surjeet Dwivedi, Jeenu Varghese, Gurpreet Kaur Walia

**Affiliations:** 1 Department of Nuclear Medicine, Command Hospital Air Force, Bengaluru, Karnataka, India; 2 Department of Surgical Oncology, Command Hospital Air Force, Bengaluru, Karnataka, India; 3 Department of Pathology, Command Hospital Air Force, Bengaluru, Karnataka, India

**Keywords:** appendicular skeletal metastasis, chronic Hepatitis B, FDG PET-CT, hepatocellular carcinoma, triple phase CECT

## Abstract

Hepatocellular carcinoma (HCC) is known to be the most common primary tumor of the liver and is also the fifth most common cancer in the world. Chronic hepatitis B and C along with type 2 diabetes mellitus and alcoholic liver disease are quite well-known risk factors for HCC, and it is uncommon in the noncirrhotic liver. HCC favors spreading as multifocal intrahepatic lesions and potential vascular invasion, and extrahepatic spread is uncommon. Skeletal metastasis from HCC occurs infrequently compared to other cancers and is common in the axial skeleton. Metastatic involvement of the appendicular skeleton is a rare entity, and the initial presentation of HCC as metastatic involvement of the appendicular skeleton is even rarer. We report a case of HCC with incidentally detected cirrhosis and chronic hepatitis B infection presenting with pain in the left shoulder.

## Introduction

Liver cancer is an increasingly significant contributor to the global cancer burden. Hepatocellular carcinoma being the principal histologic type of liver malignancy accounts for a large majority of the disease burden. The age-adjusted incidence rate of hepatocellular carcinoma (HCC) in India for men ranges from 0.7 to 7.5 and for women 0.2 to 2.2 per 100,000 of the population per year. 1 The most common risk factors for HCC worldwide include cirrhosis of the liver, HBV infection, HCV infection, alcohol consumption, and aflatoxin exposure. In addition to the above, non-alcoholic fatty liver disease (NAFLD) and diabetes mellitus are also being increasingly recognized as risk factors in the Indian population. 1 HCC generally tends to spread as multifocal intrahepatic lesions, with an increase in size and potential vascular invasion. 2 Extrahepatic metastases of HCC are known to be relatively rare at the time of initial diagnosis. 3 The most frequent site of extrahepatic metastasis are the lungs, followed by lymph nodes, bones, and adrenal gland. 4 5 The most common location of bone metastasis is the axial skeleton with the most frequently involved sites being the lumbosacral vertebrae, followed by thoracic vertebrae and cervical vertebrae. 6 We present a rather unusual case wherein an unsuspected case of HCC with incidentally detected cirrhosis and chronic hepatitis B infection initially presented with shoulder pain and was found to have an expansile lytic lesion in the scapula on further imaging.

## Case Presentation

**Fig. 1:**
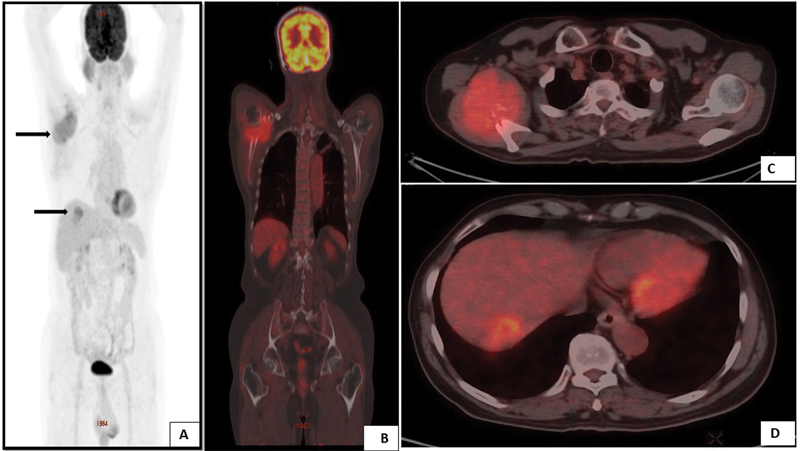
( **A** ) A maximum intensity projection image of an ^18^ F-FDG PET-CT study revealing increased tracer uptake in the right shoulder region (black arrow) along with a focal lesion in the liver ( *black arrow* ). ( **B** ) A coronal-fused ^18^ F-FDG PET-CT image showing a metabolically active expansile lytic lesion involving the right scapula. ( **C** ) An axial-fused PET-CT image showing a metabolically active expansile lytic lesion involving the right scapula. ( **D** ) An axial-fused PET-CT image showing a metabolically active hypodense lesion involving segment VII of the liver.

**Fig. 2:**
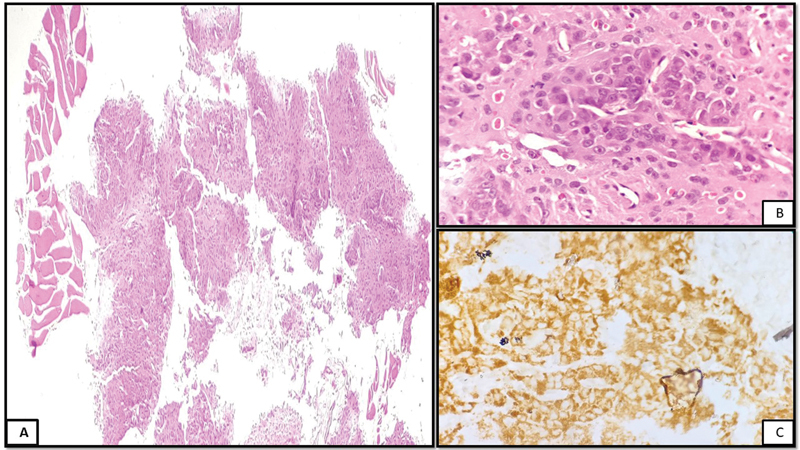
( **A** ) (hematoxylin and eosin, 40 × ) Image showing tumor arranged in thick trabeculae and sheets. ( **B** ) (hematoxylin and eosin, 400 × ) Polygonal-shaped tumor cells with moderate eosinophilic cytoplasm, pleomorphic nuclei, and prominent macro nucleoli. ( **C** ) Immunohistochemistry image (400 × ) showing strong granular cytoplasmic Hep Par 1 positivity in the tumor cells.

A 72-year-old male patient with no known comorbidities presented with a history of progressive pain in his right shoulder of 2 months duration. Radiography of the right shoulder revealed an ill-defined osteolytic lesion epicentered in the glenoid process of the right scapula. The lesion showed a poor zone of transition with chondroid matrix and no evidence of periosteal reaction. A differential diagnosis of plasmacytoma, chondrosarcoma, and metastasis was provided. A subsequent magnetic resonance imaging (MRI) scan revealed a well-defined lobulated expansile osteolytic lesion measuring 8 AP × 6 TR × 8 CC cm epicentered in the neck of the right scapula. The lesion was involving the inferolateral aspect of the spine of the scapula, upper one-third of the medial border including the infra-glenoid tubercle and glenoid process, relatively sparing the supraglenoid tubercle. The right coracoid and acromion process were spared by the lesion. T2 hyperintensity was noted in the anterosuperior aspect of the head of the right humerus; however, no obvious infiltration of the above lesion was noted. There was an associated large soft tissue component within the lesion, which appeared isointense on T1-weighted images and intermediate to high-signal intensity in T2 and proton density fat suppressed (PDFS) sequences. The lesion showed restriction of diffusion, and in the post-contrast study, there was a relatively homogenous enhancement. Based on the above findings, the differential diagnoses were metastasis, plasmacytoma, chondrosarcoma (mesenchymal variant), and telangiectatic osteosarcoma. In view of the MRI findings and a higher suspicion of metastasis, the patient was further subjected to PET-CT using ^18^ F-fluorodeoxyglucose (FDG). The PET-CT scan also revealed a metabolically active expansile lytic lesion with a soft tissue component in the right scapula. In addition, another FDG avid hypodense lesion was detected in segment VII of the liver ( Fig. 1 ). In view of the above, two differential diagnoses were provided, firstly osteosarcoma or chondrosarcoma with liver metastasis and secondly a hepatocellular carcinoma with bone metastasis in the scapula. A biopsy ( Fig. 2 ) was obtained from the osteolytic lesion in the right scapula that revealed a malignant tumor arranged in diffuse sheets and a vague acinar and trabecular pattern. The tumor cells were large, polygonal with abundant eosinophilic granular cytoplasm and prominent nucleoli with brisk mitoses one to two per high power field, and many cells also showed intranuclear inclusions. The tumor was seen infiltrating into the surrounding skeletal muscle. Areas of hemorrhage and necrosis were also noted. Immunohistochemistry revealed Hep Par 1 positivity. CK 7, CK 20, S 100, CD 68, and CD 99 were found to be negative. In view of the above, the lesion was diagnosed to be a metastatic deposit from hepatocellular carcinoma. In view of the PET-CT and biopsy findings, the patient was further subjected to a triple-phase CT with an ill-defined nonhomogenous hypodense lesion in segment VII of the liver, which was heterogeneously hyper-enhancing in the late arterial phase and showed a rapid washout in the portal venous phase. The lesion was hypoenhancing in the delayed phase. An enhancing capsule was also noted in the portal venous phase. Signs of portal hypertension were also noted in the form of a prominent portal vein (14 mm) with a few lienorenal, gastroesophageal junction, and perisplenic collaterals. On USG correlation, the liver also appeared enlarged, hyperechoic, and cirrhotic. The patient was a social drinker who used to consume less than one drink daily. Clinical examination revealed no stigmata of chronic liver disease. His laboratory workup revealed normal bilirubin levels with elevated aspartate aminotransferase/serum glutamic oxaloacetic transaminase (AST/SGOT) (80 U/L), alanine aminotransferase/serum glutamate pyruvate transaminase (ALT/SGPT) (176 U/L), and normal alkaline phosphatase levels (140 U/L). The patient was found to be HBsAg positive with elevated HBV DNA levels. Alpha feto protein (AFP) was elevated (33.85 ng/mL). Other tumor markers including carbohydrate antigen 19.9 (CA19.9) and carcinoembryonic antigen (CEA) were negative. The patient was finally diagnosed with a case of metastatic hepatocellular carcinoma in a background of cirrhosis and chronic hepatitis B with a good performance score (Eastern Cooperative Oncology Group Performance Status-1 [ECOG PS-1]). The patient was treated with radiotherapy to the bone lesion, followed by bisphosphonates, sorafenib, and entecavir.

## Discussion

It is a recognized fact that almost all kinds of cancers can spread to the bones; however, the most commonly encountered primary cancers that metastasize to the bones are from the prostate, breasts, kidneys, lungs, and thyroid. In contrast to intrahepatic metastasis, extrahepatic metastasis of HCC is relatively uncommon with a reported incidence of about 15 to 17%. 7 Risk factors for metastatic involvement especially extrahepatic metastasis include advanced tumors, index tumor size greater than 5 cm, multifocal lesions or infiltrative lesions, and vascular invasion. 8 9 The most frequent location for metastatic HCC is the lungs, followed by the lymph nodes and bones. Although bone metastases from HCC or initial presentation with bone metastasis are not very uncommon, the presentation of HCC as an isolated appendicular skeletal metastasis is extremely rare 2 with very few cases reported in the literature. The typical location of bone metastases is in the axial skeleton with the most common site being the vertebral column. Metastasis to the bones occurs through portal vein-vertebral vein plexuses, thus explaining the more frequent craniospinal and pelvic bone metastases. 10 Skeletal metastasis appears to be relatively unique among various hematogenous metastases of HCC because it can occur before clinical manifestations of chronic liver disease and is usually symptomatic, as in our present case. The pulmonary and systemic circulation is thought to be the main route of metastasis to the skeletal system.

Bone metastases from HCC usually present as expansile osteolytic lesions with soft tissue components. This phenomenon can be explained by the premetastatic niche theory where tumor-released soluble factors enter the bloodstream and induce microenvironment changes including matrix remodeling, which support subsequent cancer cell engraftment. The likely molecules involved in this mechanism include CXCL12, interleukin (IL-6), annexin II, and vascular endothelial growth factor (VEGF), as they contribute to both hematopoietic stem cell homing to the bone marrow and cancer cell infiltration and survival. In this way, cancer cells occupy the original hematopoietic stem cell niche and further colonize the site. The replacement of hematopoietic stem cells with cancer cells in the niche promotes metastatic tumor progression. 11

The histopathological diagnosis of metastatic HCC is made from cellular morphology and supporting immunohistochemistry. Although these neoplasms correspond to poorly differentiated carcinomas, they are focally trabecular with an acinar pattern. The nuclei have nuclear inclusions in the cytoplasm, and a pigment compatible with brown bile is observed. With these microscopic findings and positivity for Hep Par-1, the histopathological diagnosis of HCC is made. Hep Par-1 (human hepatocyte paraffin-1) is an antigen that reflects hepatocyte differentiation in approximately 90% of HCCs and is considered positive, the staining must be granular and cytoplasmic. 12

## Conclusion

We report a rare presentation of HCC in a background of cirrhosis caused by chronic hepatitis B infection wherein appendicular skeletal metastasis involving the scapula causing shoulder pain was the initial presentation even before any feature of chronic liver disease could be detected. Through this case report and literature review, we seek to focus on and identify the uncommon osseous metastatic sites of HCC and reappraise the role of imaging including triple-phase CT and PET-CT in detecting such rare extrahepatic sites of metastasis initially at the time of diagnosis to further guide the treating clinicians in clinching the diagnosis.
